# Anatomical features of coexisting horseshoe kidney and double inferior vena cava

**DOI:** 10.1007/s12565-025-00860-3

**Published:** 2025-06-27

**Authors:** Naofumi Horio, Narumi Miyaura, Kenta Nagahori, Daisuke Kiyoshima, Yoko Ueda, Masahito Yamamoto, Takashi Okazaki, Shogo Hayashi

**Affiliations:** 1 Tokai University School of Medicine, Isehara, Kanagawa Japan; 2 Department of Anatomy, Division of Basic Medical Science, Tokai University School of Medicine, 143, Shimokasuya, Isehara-Shi, Kanagawa 259-1193 Japan; 3 Department of Radiology, Tokai University School of Medicine, Isehara, Kanagawa Japan

**Keywords:** Double inferior vena cava, Horseshoe kidney, Multiple renal arteries, Multiple renal veins

## Abstract

To describe a rare case of coexisting horseshoe kidney (HSK) and double inferior vena cava (DIVC) observed during a cadaveric dissection course and to analyze the embryological and clinical implications of this anatomical variation. A detailed anatomical dissection of a 96-year-old Japanese male cadaver was performed during a gross anatomy course. The kidneys were fused by a fibrous and parenchymal isthmus below the inferior mesenteric artery. Four right renal arteries and three left renal arteries were identified, along with an additional renal vein arising from the isthmus and coursing to the left adrenal gland. The right and the left IVCs ascended bilaterally along the aorta, with no communicating veins observed. Histological findings revealed atrophic renal tubules in the isthmus, with increased connective tissue. This report highlights the high variability of renal and vascular anatomy in coexisting HSK and DIVC. Recognition of such variations is essential for radiologists and surgeons to avoid complications during renal transplantation, vascular surgery, and imaging interpretation. In addition, the kidneys and the IVC development occur simultaneously between weeks 4 and 8 of embryogenesis. Therefore, abnormalities of retroperitoneal structures that occur during embryological development can lead to variations.

## Introduction

Horseshoe kidney (HSK) is a well-known congenital anomaly of the upper urinary tract, characterized by the fusion of two renal masses, typically at the lower poles, connected by a renal parenchyma or fibrous isthmus crossing the midline. The prevalence of HSK is relatively low, ranging from 1 case per 400 to 800 births in Japan and Europe (Fuyuta et al. 1977; Nakamura et al 2005). Understanding the vascular anatomy of HSK is essential for radiologists and surgeons, particularly when performing procedures involving renal vasculature.

Inferior vena cava (IVC), which is formed by the union of the left and right common iliac veins, is the main venous blood channel from regions of the body below the diaphragm. IVC ascends on the right side of the abdominal aorta and drains into the right atrium (Karaosmanoglu et al. 2021). Double IVC (DIVC) is a rare anatomical variation in which two IVCs run bilaterally parallel to the abdominal aorta. Its global prevalence is estimated to range from 0.2 to 3%, and it is often identified incidentally during retroperitoneal surgery, imaging studies, or cadaveric dissection (Cheung and Wong 2020). Variations involving the left IVC, renal veins (RVs), and gonadal veins in DIVC carry significant clinical implications, particularly in procedures, such as renal transplantation and laparoscopic adrenalectomy (Radermecker et al. 2008).

We report the discovery of coexisting HSK and DIVC during routine dissection by medical students. This report provides the detailed anatomy and histology of these variations and the associated vascular system. Additionally, we discuss the clinical and the embryological significance of these variations.

## Case report

HSK and DIVC were discovered in 96-year-old Japanese male cadaver during dissection by students enrolled in a gross anatomy course at Tokai University in 2023.

### HSK

**Fig. 1:**
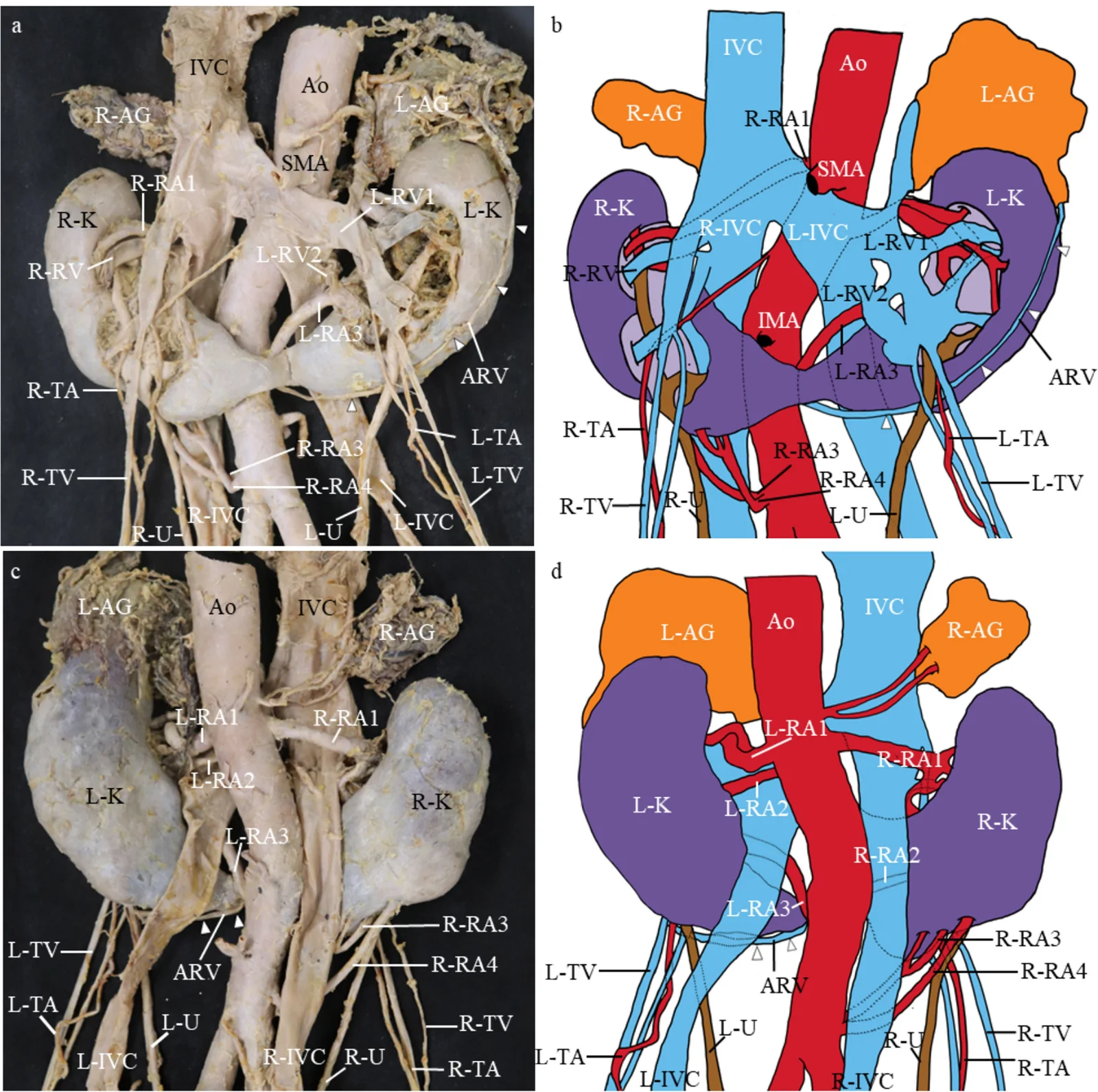
Anterior and posterior views of horseshoe kidney (HSK) and double inferior vena cava (DIVC). **a** Anterior showing the HSK and bilateral IVCs. **b** Corresponding schematic of (**a**) highlighting the surrounding anatomical structures. **c** Posterior view of the HSK and DIVC. **d** Schematic of (**c**) with labeled surrounding structures. White arrowheads indicate the additional renal vein (ARV) tract. Renal arteries and veins are numbered sequentially from a cranial to caudal direction. *Ao* aorta, *AG* adrenal gland, *IMA* inferior mesenteric artery, *IVC* inferior vena cava, *K* kidney, *L* left, *R* right, *RA* right renal artery, *RV* renal vein, *SMA* superior mesenteric artery, *TA* testicular artery, *TV* testicular vein, *U* ureter

Left and right kidneys were located between the middle region of T12 and the lower region of L2. The isthmus connected the lower poles of both kidneys at the lower region of L2 below the origin of the inferior mesenteric artery (IMA) (Fig. 1a, b). Both renal hills were widely open toward the ventral direction. Both ureters originated independently from each renal pelvis and descended, thus forming shallow pressure grooves on the ventral surface of the kidney and terminated at the bladder in their usual anatomical position.

### DIVC

Right and left common iliac veins formed at the level of the L5 vertebra from the junction of the ipsilateral internal and external iliac veins. These common iliac veins did not join at the L4 vertebral level to form the IVC but ran bilaterally toward the abdominal aorta and transitioned into left and right IVC (Fig. 1a, c). The right IVC ascended along the right side of the aorta, received the right RV, and continued superiorly to the end at the inferior part of the right atrium. The left IVC ascended cranially along the left side of the aorta and received two left RVs (Fig. 1b). The left IVC continued with the major preaortic trunk, which crossed the anterior of the aorta that traveled obliquely and emptied into the right IVC. Notably, no communicating veins were observed between right and left IVCs (Fig. 1c, d).

### Renal arteries (RA)

Four right RAs (R-RAs) arose from the aorta and supplied the right kidney. The first R-RA (R-RA1) emerged from the right surface of the aorta at the level of the superior mesenteric artery (SMA) and diverged into two branches supplying the apical and upper regions (Fig. 1a, c). The second R-RA (R-RA2) arose from the right ventrolateral side of the aorta below the IMA, coursed posterior to the isthmus and right kidney, and distributed to the posterior middle region (Fig. 1d). The third R-RA (R-RA3) arose from the right dorsolateral side of the aorta above the common iliac artery, proceeded anterior to the right IVC, and supplied the lower posterolateral region. The final R-RA (R-RA4) originated below the R-RA3 and divided into three branches supplying the lower renal hilum and lower kidney (Fig. 1).

Three left RAs (L-RAs) also arose from the aorta to supply the left kidney. The first L-RA (L-RA1) arose from the left surface of the aorta below the origin of the SMA, divided into four branches, and supplied the apical, the upper, and the middle regions. The second L-RA (L-RA2) arose below the SMA, coursed posterior to the left renal vein, and supplied the upper kidney. The third L-RA (L-RA3) originated below the IMA, posterior to the isthmus, and entered the middle and lower renal hilum.

### RVs

Four RVs were identified. The right RV (R-RV) drained into the right IVC at approximately the middle level of L1 and coursed along the ventral side of R-RA1. It received blood from the apical, the upper, the middle, and the posterior regions of the right kidney, as well as the right testicular and adrenal veins (Fig. 1). On the left, two RVs entered the left IVC separately. The superior left RV (L-RV1) drained at the upper level of L1, ventral to L-RA1, while the inferior left RV (L-RV2) drained at the lower level of L1. These two veins formed a loop in front of the left renal hilum and collected blood from the apical, the upper, the middle, and the posterior kidney regions, along with the left testicular veins. An additional renal vein (ARV), 1 mm in diameter, emerged from the isthmus, ascended laterally along the left kidney, and drained into the left adrenal gland (Fig. 1a, b).

### Other arteries

Celiac trunk, SMA, and IMA arose from the ventral side of the aorta. The right inferior phrenic artery originated from the celiac trunk, while the left inferior phrenic artery arose directly from the aorta. The left superior adrenal arteries arose from the left inferior phrenic artery, and a single left middle adrenal artery arose directly from the aorta. The left inferior adrenal artery was absent, and no adrenal artery originated from the RAs. The right testicular artery originated from the aorta while the left emerged from L-RA. Both testicular arteries coursed ventrally across the kidney (Fig. 1).

### Histology

**Fig. 2:**
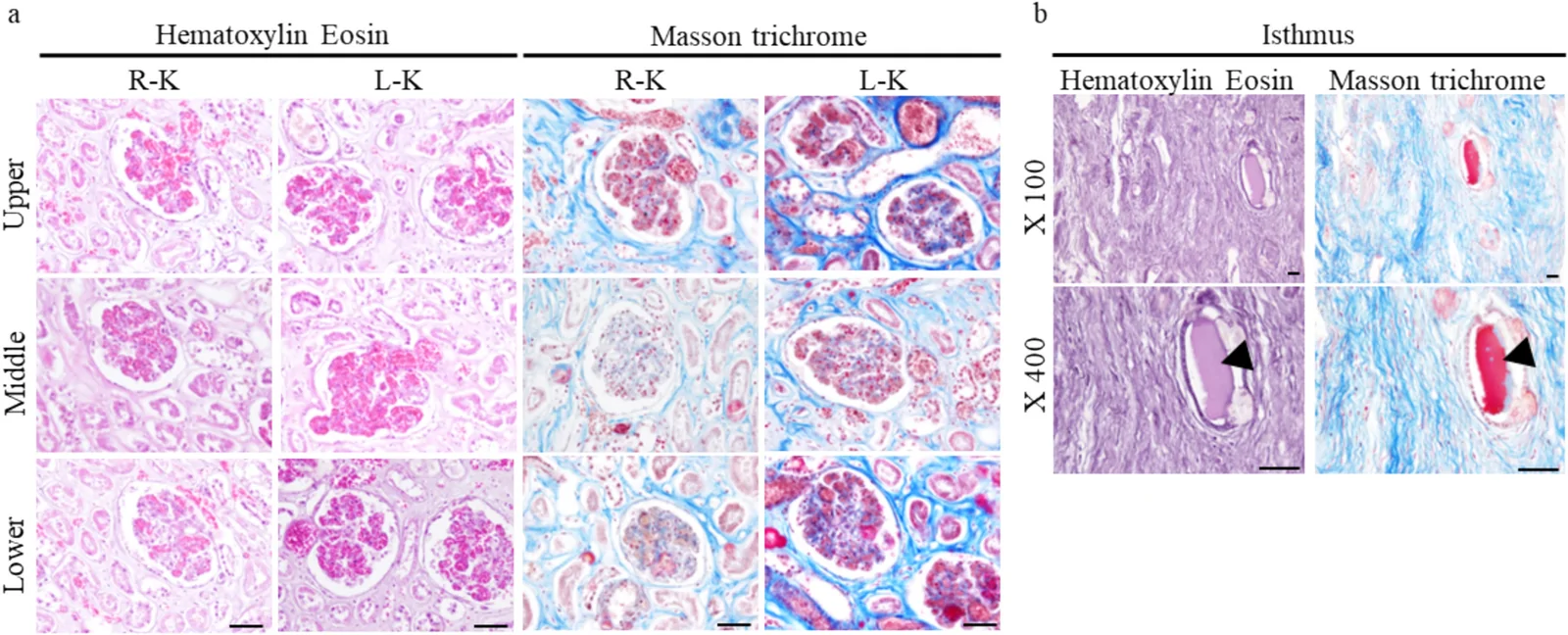
Histological findings of horseshoe kidney. **a** Sections from the right and the left kidneys stained with hematoxylin eosin and Masson’s trichrome (original magnification, × 400) show normal renal parenchymal structures. Scale bar = 50 µm **b** Histological analysis of the isthmus, with tissue removed from its dorsal side and stained with hematoxylin eosin and Masson’s trichrome. Black arrowheads indicate expanded renal tubules containing eosinophilic material. *R-K* right kidney, *L-K* left kidney. Scale bar = 50 µm

The right and the left kidneys exhibited a normal renal parenchymal structure, with the cortex containing numerous renal corpuscles and tubules, indicating preserved renal function (Fig. 2a). In contrast, the proportion of connective tissue in the isthmus tended to be higher than that in the right and the left kidneys. The glomerular structure was not found, and the urinary tubules were atrophic or greatly dilated, lined by flattened epithelium, and filled with eosinophilic material (so-called thyroidization) (Fig. 2b).

## Discussion

According to Takemoto et al. (1978), DIVC can be classified into six types. The variation observed in our case was type IIb-2 because the diameters of the right and left IVCs were nearly identical, and no communicating veins were present. HSK is known to exhibit significant arterial variations (Glodny et al. 2009). Usually, the kidney develops from the metanephros in mammals and the metanephros appears in the pelvis, at the level of S1–S2, in the 28th–32nd days of gestation (Woolf et al. 2003). Between the 6th and 9th week of gestation, the kidneys rotate medially and ascend into the retroperitoneal space from the pelvic to the lumbar level. Upon this evolution, the mesonephros is supplied on either side by a maximum of 30 mesonephric arteries (Felix 1912). These arteries are temporary aortic branches, and all but two of the mesonephric arteries retract, leaving one artery on each side at the second lumbar level. In this case, the right and the left kidneys were supplied by four and three arteries, respectively. Felix (1912) speculated that the metanephros climb upward, like climbing a ladder. Therefore, it was estimated that these additional arteries were remnants of the mesonephric arteries. Furthermore, two L-RVs drained into the left IVC, and an ARV arose from the isthmus and drained into the left adrenal gland. While previous studies have reported ARVs draining into the common iliac vein (Karaosmanoglu et al. 2021), no reported cases have described an ARV proceeding to the adrenal gland. The vascular communication between the perirenal veins and the vessels derived from the adrenal gland has a course similar to that of the ARV in this case; however, since the ARV originates from the kidney, it is considered separate from the perirenal vein. Generally, these vessels and the RV, including the ARV, are separated by tissues, such as the renal fascia, and are rarely directly connected (Ochi et al. 2020). HSK is known to fuse when the two posterior poles come close to each other at 5–7 weeks of embryogenesis, in the absence of a defining fascia or adipose tissue (Humphries et al. 2023). Therefore, abnormalities in the fusion of the posterior poles may affect the separation between the tissues, such as the renal fascia, as well as connective tissue continuity, potentially affecting venous development. Thus, the coexistence of HSK and DIVC results in complex and unexpected vascular anatomy. Given that patients with HSK are predisposed to hydronephrosis due to blood flow stagnation, we hypothesize that the additional veins may play a compensatory role in preventing hydronephrosis development, as demonstrated by symptom improvement following isthmusectomy (Jarzemski and Listopadzki 2014).

The incidence of IVC variations is higher in patients with HSK than those without (Ichikawa et al. 2011,). Kidneys and IVC develop simultaneously between weeks 4 and 8 of embryogenesis during which the kidneys ascend from the pelvis and undergo rotation. The ascent of HSK is arrested when the isthmus becomes trapped under the IMA, leading to its lower-than-usual position in the anterior pelvis (Basso 2011). Simultaneously, IVC formation involves the anastomosis and the degeneration of posterior cardinal, subcardinal, and supracardinal veins (Chen et al. 2012). In this case, supracardinal and subcardinal veins remained on the left side of the anastomosis. Furthermore, ARV is considered a remnant of the subcardinal anastomosis during embryological development of the IVC. HSK and DIVC development occurred simultaneously between weeks 4 and 8 of embryogenesis. Therefore, abnormalities in the fusion and rotation of HSK may have a direct effect on venous development.

This report adds to the limited number of documented cases of coexisting HSK and DIVC (Table 1). Unlike Adachi’s earlier report, which described a communicating vein between the right and the left IVCs and single renal arteries on each side with an isthmic branch (Adachi 1940), our case demonstrated multiple renal arteries and the absence of any communicating veins (Table 1). These findings underscore a high variability in the coexistence of HSK and DIVC. Furthermore, the present coexistence of HSK and DIVC or left IVC has been reported only eight times in clinical cases (Ichikawa et al. 2011; Radermecker et al. 2008; Sonneveld et al. 1999). According to these reports, HSK and abnormalities of IVC are usually asymptomatic and were discovered incidentally on contrast-enhanced spiral computed tomography before surgery such as open aortic abdominal aneurysm repair. Therefore, recognition of such anatomical variations is critical for renal transplantation, vascular surgeries, and other surgical procedures to avoid complications. Understanding these anomalies also enhances awareness of developmental processes and their potential clinical implications.

**Table 1:** Comparison of the present case with previous cases

	Population and number of cases	Morphological types of HSK	DIVC classification (Takemoto et al.’s classification.)	Associated renal vascular/other vascular anomalies
Our case	Japanese, 1 cadaveric case	The HSK is symmetrical, with a midline fusion at the lower pole. The isthmus has a fibrotic tissue and is rather slender	TypeIIb-1: In our case, the left and right IVC are also well developed, but the communicating vein is not found	Total 7 RAs: 4 right and 3 left RAs, all seen arising from the aorta Total 4 RVs: 1 right, 2 left RVs, and an additional renal vein from the isthmus, right and left RVs seen draining into each IVC. The additional renal vein ascended laterally along the left kidney and drained into the left adrenal gland
Adachi (1940)	Japanese, 1 cadaveric case	The HSK is symmetrical, with a midline fusion at the lower pole. The isthmus has a functional renal parenchyma and is relatively thick	TypeIIb-3: In Adachi’s case, the left and right IVC are also well developed, and the communicating vein or anastomosis is relatively thick	Total 3 RAs: 1 right, 1 left RAs and an additional artery to the isthmus, all seen arising from the aorta Total 3 RVs: 1 right, 1 left RVs and an additional vein from the isthmus, the right RV seen draining into the right IVC. The left RV and additional renal vein drained into the left IVC

*DIVC* double inferior vena cava, *HSK* horseshoe kidney, *IVC* inferior vena cava, *RA* renal artery, *RV* renal vein

## Data Availability

No datasets were generated or analyzed during the current study.

## References

[CR1] Adachi B (1940) Das venensystem der Japaner. Anatomic der Japaner, vol 2. Kenkyusha, Kyoto

[CR2] Basso LS (2011) Abnormal vascular supply of the horseshoe kidney: case report and review of the literature. Anatomy 5:48–52

[CR3] Chen H, Emura S, Nagasaki S, Kubo KY (2012) Double inferior vena cava with interiliac vein: a case report and literature review. Okajimas Folia Anat Jpn 88:147–15122645906 10.2535/ofaj.88.147

[CR4] Cheung KT, Wong E (2020) Duplicated inferior vena cava in a patient with ampullary adenocarcinoma: A case report and literature review of anatomical variations. Cureus 12:e1157633364101 10.7759/cureus.11576PMC7749830

[CR5] Felix W (1912) The development of the urogenital organs. In: Keibel F, Mall FP (eds) Manual of human embryology. Lippincott Publishers Company, Philadelphia, J. B, pp 752–880

[CR6] Fuyuta M, Ukeshima A, Miyayama Y (1977) An autopsy case of horseshoe kidney. Okajimas Folia Anat Jpn 53:323–326859711 10.2535/ofaj1936.53.6_323

[CR7] Glodny B, Petersen J, Hofmann KJ et al (2009) Kidney fusion anomalies revisited: clinical and radiological analysis of 209 cases of crossed fused ectopia and horseshoe kidney. BJU Int 103:224–23518710445 10.1111/j.1464-410X.2008.07912.x

[CR8] Humphries A, Speroni S, Eden K, Nolan M, Gilbert C, McNamara J (2023) Horseshoe kidney: Morphologic features, embryologic and genetic etiologies, and surgical implications. Clin Anat 36:1081–108836708162 10.1002/ca.24018

[CR9] Ichikawa T, Kawada S, Koizumi J et al (2011) Major venous anomalies are frequently associated with horseshoe kidneys. Circ J 75:2872–287722001291 10.1253/circj.cj-11-0613

[CR10] Jarzemski P, Listopadzki S (2014) Laparoscopic horseshoe kidney isthmusectomy: four case reports. Wideochir Inne Tech Maloinwazyjne 9:115–12024729821 10.5114/wiitm.2011.35740PMC3983536

[CR11] Karaosmanoglu AD, Ardali Duzgun S, Akata D, Ozmen MN, Karcaaltincaba M (2021) A sling of a normal right inferior vena cava around the abdominal aorta. Surg Radiol Anat 43:1391–139433547916 10.1007/s00276-021-02693-x

[CR12] Nakamura Y, Yi SQ, Iimura A, Terayama H, Naito M, Itoh M (2005) Morphological observation of the horseshoe kidney with special reference to the vascular system in 2 Japanese cadavers. Okajimas Folia Anat Jpn 82:89–9416350421 10.2535/ofaj.82.89

[CR13] Ochi A, Muro S, Adachi T, Akita K (2020) Zoning inside the renal fascia: The anatomical relationship between the urinary system and perirenal fat. Int J Urol 27:625–63332314429 10.1111/iju.14248PMC7384158

[CR14] Radermecker MA, Van Damme H, Kerzmann A, Creemers E, Limet R (2008) Association of abdominal aortic aneurysm, horseshoe kidneys, and left-sided inferior vena cava: report of two cases. J Vasc Surg 47:645–64818295119 10.1016/j.jvs.2007.08.053

[CR15] Sonneveld DJ, Van Dop HR, Van der Tol A (1999) Resection of abdominal aortic aneurysm in a patient with left-sided inferior vena cava and horseshoe kidney. J Cardiovasc Surg (Torino) 40(3):421–424 10412933

[CR16] Takemoto R, Tezuka M, Yada D (1978) Four cases of anomalous postrenal vena cava with special regard to the classification of this kind of anomaly (author’s transl). Kaibogaku Zasshi 53:423–434 (**Japanese**) 742345

[CR17] Woolf AS, Winyard PJD, Hermanns MH, Welham SJM (2003) Maldevelopment of the human kidney and lower urinary tract: an overview. Development of kidney blood vessels. In: Vize PD, Woolf AS, Bard JBL (eds) The kidney: from normal development to congenital disease. Academic Press, China, pp 377–393

